# Opportunities for penicillin allergy evaluation in dental clinics

**DOI:** 10.1017/ash.2022.18

**Published:** 2022-04-11

**Authors:** Amanda Vivo, Michael J. Durkin, Ibuola Kale, Taylor Boyer, Margaret A. Fitzpatrick, Charlesnika T. Evans, M. Marianne Jurasic, Gretchen Gibson, Katie J. Suda

**Affiliations:** 1 Center of Innovation for Complex Chronic Healthcare, Edward Hines Jr. Veterans’ Affairs (VA) Medical Center, Hines, Illinois; 2 Washington University School of Medicine, St Louis, Missouri; 3 Center for Health Equity Research and Promotion, VA Pittsburgh Health Care System, Pittsburgh, Pennsylvania; 4 Loyola University Chicago Stritch School of Medicine, Maywood, Illinois; 5 Northwestern University Feinberg School of Medicine, Chicago, Illinois; 6 Veterans’ Health Administration Office of Dentistry, Washington, DC; 7 Boston University Henry M. Goldman School of Dental Medicine, Boston, Massachusetts; 8 Center for Healthcare Organization and Implementation Research, VA Bedford Healthcare System, Bedford, Massachusetts; 9 Department of Medicine, School of Medicine, University of Pittsburgh, Pittsburgh, Pennsylvania

## Abstract

**Objective:**

To evaluate opportunities for assessing penicillin allergies among patients presenting to dental clinics.

**Design:**

Retrospective cross-sectional study.

**Setting:**

VA dental clinics.

**Patients:**

Adult patients with a documented penicillin allergy who received an antibiotic from a dentist between January 1, 2015, and December 31, 2018, were included.

**Methods:**

Chart reviews were completed on random samples of 100 patients who received a noncephalosporin antibiotic and 200 patients who received a cephalosporin. Each allergy was categorized by severity. These categories were used to determine patient eligibility for 3 testing groups based on peer-reviewed algorithms: (1) no testing, (2) skin testing, and (3) oral test-dose challenge. Descriptive and bivariate statistics were used to compare facility and patient demographics first between true penicillin allergy, pseudo penicillin allergy, and missing allergy documentation, and between those who received a cephalosporin and those who did not at the dental visit.

**Results:**

Overall, 19% lacked documentation of the nature of allergic reaction, 53% were eligible for skin testing, 27% were eligible for an oral test-dose challenge, and 1% were contraindicated from testing. Male patients and African American patients were less likely to receive a cephalosporin.

**Conclusions:**

Most penicillin-allergic patients in the VA receiving an antibiotic from a dentist are eligible for penicillin skin testing or an oral penicillin challenge. Further research is needed to understand the role of dentists and dental clinics in assessing penicillin allergies.

Penicillin allergy is the most commonly reported drug allergy in the United States^1,2^; ∼10% of the population (32 million people in the United States) report a penicillin allergy.^2–4^ However, 90% of those reporting a penicillin allergy are not truly allergic. Rather, most patients with a labeled allergy may have an intolerance to penicillin, another cause of symptoms thought to have been an allergic reaction to penicillin, or had their allergy wane over time.^3,5^

Inaccurate or outdated penicillin allergy documentation has important real-world consequences.^6,7^ Patients with a labeled penicillin allergy are more likely to receive broad-spectrum antibiotics, which contributes to antibiotic resistance both at a patient and societal level. Countries with higher antibiotic use have higher rates of antibiotic resistance, and individuals who receive broad-spectrum antibiotics are more likely to acquire antibiotic-resistant infections. Indeed, penicillin allergic patients are at a 26% increased risk of *C. difficile* infection and 69% increased risk of methicillin-resistant *S. aureus* infections (MRSA) compared to matched patients without a penicillin allergy.^8^ Furthermore, patients undergoing surgery who receive prophylaxis with a second-line antibiotic due to a penicillin allergy were reported to have a 50% increased odds of surgical site infections compared to those without a reported penicillin allergy.^9^

Formal penicillin allergy evaluation programs are becoming more common in inpatient and outpatient settings. Standardized penicillin allergy algorithms make a penicillin allergy evaluation straightforward.^6,10^ This trend has expanded the opportunity for penicillin allergy evaluations to be conducted by emergency clinicians, internists, infectious disease specialists, and pharmacists.^10–17^ However, such programs still require an initial interaction with medical clinicians in hospital or clinic settings and, thus, are generally not accessible to people who do not access medical care. Many patients, particularly young individuals with few medical problems or individuals with no medical problems, do not regularly seek care from medical clinicians, but may see a dentist.^18,19^

Dental offices may serve as an important place for a penicillin allergy evaluation. The most commonly prescribed antibiotic by dentists is amoxicillin, and penicillin-based antibiotics remain first-line agents for essentially all dental antibiotic use.^20^ Furthermore, cephalexin, a first-generation cephalosporin, is included in dental guidelines as a reasonable alternative for those with nonsevere penicillin allergies.^21–23^ The primary objective of this study was to identify the rate of true penicillin allergy among patients receiving dental care and to evaluate how many patients would be eligible for skin testing or oral penicillin challenge. The secondary objective was to identify the frequency of allergic reactions and explore differences in characteristics in penicillin-allergic patients who received a cephalosporin.

## Methods

### Study design and setting

This retrospective cross-sectional analysis of national Veterans’ Affairs (VA) data included adult patients (aged ≥18 years) with an outpatient dental clinic visit between 2015 and 2018. From this cohort, patients who received an antibiotic prescribed by a dentist within 7 days before or after the dental visit and with a penicillin allergy prior to the antibiotic dispense date were identified. A penicillin allergy was defined as clinician chart documentation of an “allergic reaction” to penicillin in the Corporate Data Warehouse (CDW) medication allergy domain, or an *International Classification of Disease, Ninth* or *Tenth Revision* (ICD-9/10) combined with E codes indicating a penicillin allergic reaction (Supplementary Table 1 online). Allergic reactions were identified from 1992 (oldest data available) until the prescription dispense date. Patients with a history of anaphylaxis (ie, had an ICD-9/10 code for anaphylaxis) to penicillin were excluded because it is considered a high-risk reaction, and these patients should only have their allergy evaluated by an allergy or immunology specialist.^10^ Patients with a documented oral infection using ICD-9/10 codes 7 days prior to the dental visit were excluded, consistent with prior research.^24^ The remaining penicillin allergic patients receiving a dental antibiotic were categorized into 2 patient cohorts: those who received a noncephalosporin antibiotic and those who received a cephalosporin antibiotic. Each cohort then underwent stratified random sampling by geographic region, so that each region had the same number of patients. Cohort 1 comprised patients with a penicillin allergy who received a noncephalosporin antibiotic (n = 100; 25 per region). Cohort 2 comprised patients with a penicillin allergy who received a cephalosporin (n = 200; 50 per region) (Supplementary Fig. 1 online). The group of patients with a documented penicillin allergy who received a cephalosporin were intentionally oversampled due to the infrequent number of these cases and to capture postcephalosporin allergic reactions.

**Table 1.** Penicillin Allergy Histories for Penicillin-Allergic Patients Who Did Not Receive Cephalosporin

Variable	Overall (N = 100)^a^	Pseudo Penicillin Allergy(N = 10)	True Penicillin Allergy(N = 71)	*P* Value
**Penicillin intolerance histories^c^**				
Isolated GI upset (diarrhea, nausea, vomiting, abdominal pain)	6 (6.0)	3 (30.0)	3 (4.2)	.0184^b^
Other	15 (15.0)	2 (20.0)	13 (18.3)	.0908^b^
**Low-risk allergy histories^d^**				
Itching (pruritus)	7 (7.0)	1 (10.0)	6 (8.5)	.4326^b^
Unknown, remote (<10 y prior)	5 (5.0)	4 (40.0)	1 (1.4)	**.0004** ^b^
**Moderate-to-high-risk allergy histories^e^**				
Anaphylaxis	14 (14.0)	0 (0)	14 (19.7)	.0296^b^
Throat tightness	1 (1.0)	1 (1.0)	0 (0)	1.0000^b^
Shortness of breath	4 (4.0)	0 (0)	4 (5.6)	.7231^b^
Angioedema/swelling	8 (8.0)	0 (0)	8 (11.3)	.2677^b^
Hypotension	1 (1.0)	0 (0)	1 (1.4)	1.0000^b^
Rash	57 (57.0)	0 (0)	57 (80.3)	**<.0001**
Dizzy/lightheadedness	1 (1.0)	0 (0)	1 (1.4)	1.0000^b^
Bronchospasm	2 (2.0)	0 (0)	2 (2.8)	1.0000^b^
Arrythmia	2 (2.0)	0 (0)	2 (2.8)	1.0000^b^
Flushing/redness	1 (1.0)	0 (0)	1 (1.4)	1.0000^b^
Syncope/pass out	1 (1.0)	0 (0)	1 (1.4)	1.0000^b^
**High-risk histories^f^**				
Fever	1 (1.0)	0 (0)	1 (1.4)	1.0000^b^

Note. Some patients had multiple reactions. Significant results (*P* < .0007, Bonferroni corrected) are shown in bold.

a 19 patients did not have a specific allergic reaction documented in their electronic medical record and are not shown in the table, as cell frequencies would be zero.

b Fisher exact test.

c These penicillin intolerance histories had frequencies of 0: chills (rigors), headache, fatigue.

d These low-risk allergy histories had frequencies of 0: family history, patient denies allergy but is on record.

e These moderate- to high-risk allergy histories had frequencies of 0: cough, wheezing, nasal symptoms.

f These high-risk histories had frequencies of 0: Stevens-Johnson, dystonia, drug reaction eosinophilia, serum sickness, organ injury (liver/kidney), anemia, thrombocytopenia, erythema multiforme, acute generalized exantematous.

Data were extracted from the VA CDW, a national repository that includes clinical and administrative data from the Veterans’ Health Administration (VHA). These data are updated on a continual basis and were used to obtain patient demographics, comorbidities, facility, and dental visit characteristics. Patient demographics included sex, race, age, smoking status, and antibiotic allergy status. Dental visit information was also extracted, including procedure type, and antibiotic associated with the visit. Visits could have >1 procedure type and >1 antibiotic prescription associated with that visit. Antibiotic hypersensitivity 14 and 30 days after the antibiotic prescription date using ICD-9/10 code and E codes were extracted, as well as anaphylaxis 14 and 30 days after the antibiotic prescription date. Antibiotic hypersensitivity was defined as ICD-9/10 codes for dermatitis due to drug, allergic urticaria, angioneurotic edema, and anaphylaxis. Anaphylaxis was defined as ICD-9/10 codes for anaphylaxis.^25,26^ The definition of invasive procedures that involved manipulation of the gingiva was consistent with past work and American Heart Association/American Dental Association (ADA/AHA) guidelines.^27^ Facility characteristics included rurality, facility complexity, and geographic region. Facility complexity levels were based on patient characteristics, clinical programs, and teaching programs and categorized as high complexity (1a–c), moderate complexity (2), and low complexity (3). Geographic region was based on US Census Bureau categories. Comorbidities were identified using ICD-9/10 codes in the 365 days prior to the dental visit and were used to calculate the Charlson comorbidity index, Gagne combined comorbidity score, and Elixhauser comorbidity index. The definitions of cardiac conditions and prosthetic orthopedic implant were consistent with past work and AHA/ADA guidelines.^27^

Manual review of the electronic health record identified the dental provider specialty and documented signs and symptoms of the penicillin allergic reaction (if available). More than 1 dental provider and allergic reaction could be associated with each visit or patient. Dental residents may have been involved in the visit, but the extent of supervision was not documented in the visit note. Each allergy reaction was then categorized into intolerance, low risk, moderate-to-high risk, and high risk based on published penicillin allergy evaluation algorithms by Shenoy et al^10^ (Supplementary Table 2 online). These intolerances, low-risk, moderate-risk, and high-risk categories were then used to quantify the number of patients who were eligible for oral penicillin test challenge, those who would be eligible for penicillin skin testing, and those for whom penicillin allergy testing would be contraindicated. A high-risk reaction would indicate that the patient is contraindicated for allergy testing. A moderate-to-high-risk reaction would indicate that the patient is eligible for skin testing. Penicillin intolerance or low-risk reaction indicates that the patient is eligible for skin or oral testing. Patients with multiple allergic reactions to penicillin were categorized by the most severe allergy (eg, a patient with both a low-risk and high-risk allergy to penicillin were considered high risk). Based on the findings of Shenoy et al,^10^ patients were then placed into the following allergy categories: true penicillin allergy, pseudo penicillin allergy (defined as intolerance and low risk by Shenoy et al), and missing allergic reaction documentation (Supplementary Table 2 online). A true allergy was defined as moderate-to-high-risk or high-risk allergic reaction to penicillin. Pseudo penicillin allergy was defined as penicillin intolerances or low-risk allergic reactions to penicillin. Missing allergic reaction documentation was defined as “allergic reactant” to penicillin using CDW medication allergy domain. In this case, a clinician entered a reaction to penicillin into their medical record, but ICD-9/10 codes were missing for dermatitis due to drug, allergic urticaria, angioneurotic edema, and anaphylaxis as were E codes. The institutional review board at the Edward Hines, Jr, VA Hospital and VA Pittsburgh Healthcare System approved this study.

**Table 2.** Patient Demographics and Facility Characteristics for Penicillin-Allergic Patients who Did Not Receive a Cephalosporin Antibiotic

Variable	Overall (N = 100)	Reaction not Specified in Chart/Missing (N = 19)	Pseudo Penicillin Allergy (N = 10)	True Penicillin Allergy (N = 71)	*P* Value
**Patient demographics and characteristics**					
**Sex**					.8538^a^
Female	8 (8.0)	1 (5.3)	0 (0)	7 (9.9)	
Male	92 (92.0)	18 (94.7)	10 (100.0)	64 (90.1)	
**Race**					.7951^a^
African American	24 (24.0)	5 (26.3)	3 (30.0)	16 (22.5)	
White	70 (70.0)	12 (63.2)	7 (70.0)	51 (71.8)	
Other	1 (1.0)	0 (0)	0 (0)	1 (1.4)	
Missing	5 (5.0)	2 (10.5)	0 (0)	3 (4.2)	
**Age**					.4336^a^
18–44 y	10 (1.0)	0 (0)	1 (10.0)	9 (12.7)	
45–64 y	31 (31.0)	8 (42.1)	2 (20.0)	21 (29.6)	
>64 y	59 (59.0)	11 (57.9)	7 (70.0)	41 (57.7)	
**Smoking**					.8462^a^
Current smoker	28 (28.0)	5 (26.3)	4 (40.0)	19 (26.8)	
Never smoked	17 (17.0)	4 (21.1)	0 (0)	13 (18.3)	
Past smoker	23 (23.0)	4 (21.0)	2 (20.0)	17 (23.9)	
Missing	32 (32.0)	6 (31.6)	4 (40.0)	22 (31.0)	
**Antibiotic allergies**					
β-lactams	1.0 (1.0)	0 (0)	0 (0)	1 (1.4)	1.0000^a^
Cephalosporins	5 (5.0)	0 (0)	0 (0)	5 (7.0)	.7548^a^
Sulfas	14 (14.0)	0 (0)	1 (10.0)	13 (18.3)	.0989^a^
Macrolides	4 (4.0)	0 (0)	0 (0)	4 (5.6)	.7231^a^
Quinolones	9 (9.0)	3 (15.8)	0 (0)	6 (8.5)	.5126^a^
Other	10 (10.0)	2 (10.5)	1 (10.0)	7 (9.9)	1.0000^a^
**Comorbidities**					
Cardiac condition	25 (25.0)	8 (42.1)	3 (30.0)	14 (19.7)	.1137 ^a^
Prosthetic orthopedic implant	31 (31.0)	8 (42.1)	2 (20.0)	21 (29.6)	.4214
HIV-AIDS	0 (0)	0 (0)	0 (0)	0 (0)	…
Cancer	4 (4.0)	1 (5.3)	1 (10.0)	2 (2.8)	.3295^a^
Heart failure	7 (7.0)	3 (15.8)	0 (0)	4 (5.6)	.2600^a^
Chronic pulmonary disease	11 (11.00)	2 (10.5)	0 (0)	9 (12.7)	.6806^a^
Cerebrovascular disease	2 (2.0)	1 (5.3)	0 (0)	1 (1.4)	.4980^a^
Dementia	4 (4.0)	1 (5.3)	0 (0)	3 (4.2)	1.0000^a^
Diabetes	11 (11.00)	5 (26.3)	0 (0)	6 (8.5)	.0673^a^
Liver disease	4 (4.0)	1 (5.3)	0 (0)	3 (4.2)	1.0000^a^
Metastatic tumor	0 (0)	0 (0)	0 (0)	0 (0)	…
Myocardial infarction	0 (0)	0 (0)	0 (0)	0 (0)	…
Paralysis	0 (0)	0 (0)	0 (0)	0 (0)	…
Peripheral vascular disease	2 (2.0)	2 (10.5)	0 (0)	0 (0)	.0436^a^
Renal disease	10 (10.0)	3 (15.8)	1 (10.0)	6 (8.5)	.5650^a^
Rheumatic disease	1 (1.0)	0 (0)	0 (0)	1 (1.4)	1.0000^a^
Peptic ulcer disease	1 (1.0)	0 (0)	0 (0)	1 (1.4)	1.0000^a^
Gagne score median, mean (SD)	3, 9 (14.0)	9, 15 (18.6)	2, 8 (12.6)	1, 7 (12.4)	.0418^b^
Charlson score median, mean (SD)	0, 1 (1.6)	0, 1 (2.3)	0, 0 (1.3)	0, 1 (1.5)	.2856^b^
Elixhauser median, mean (SD)	2, 2 (2.1)	2, 3 (3.0)	2, 2 (2.1)	1, 2 (1.7)	.0459^b^
**Facility characteristics**					
**Rural**					.3954^a^
No	80 (80.0)	15 (78.9)	9 (90.0)	56 (78.9)	
Yes	19 (19.0)	3 (15.8)	1 (10.0)	15 (21.1)	
Missing	1 (1.0)	1 (5.3)	0 (0)	0 (0)	
**Complexity**					.6079^a^
1a	38 (38.0)	7 (36.8)	2 (20.0)	29 (40.8)	
1b	24 (24.0)	4 (21.1)	3 (30.0)	17 (23.9)	
1c	16 (16.0)	4 (21.1)	2 (20.0)	10 (14.1)	
2	8 (8.0)	0 (0)	2 (20.0)	6 (8.4)	
3	14 (14.0)	4 (21.0)	1 (10.0)	9 (12.7)	
**US Census region**					.3193^a^
Midwest	25 (25.0)	4 (21.0)	3 (30.0)	18 (25.4)	
Northeast	25 (25.0)	3 (15.8)	4 (40.0)	18 (25.4)	
South and other	25 (25.0)	8 (42.1)	0 (0)	17 (23.9)	
West	25 (25.0)	4 (21.1)	3 (30.0)	18 (25.4)	

Note. Statistical significance = *P* < .0007, Bonferroni corrected.

a Fisher exact test.

b Kruskal-Wallis test.

### Statistical analyses

Descriptive and bivariate statistics, including global χ^2^, the Fisher exact test, and the Kruskal-Wallis test, were used to summarize and compare facility and patient demographics between those with a pseudo penicillin allergy, those with a true penicillin allergy, and those who were missing documentation of the type of allergic reaction in cohort 1. A Bonferroni correction was applied, and the level of significance was set at *P* < .0007. Unadjusted odds ratios (ORs) and 95% confidence intervals (CIs) were used to calculate the odds of a specific characteristic being associated with a cephalosporin antibiotic prescription. SAS version 9.4 software (SAS Institute, Cary, NC) was used for data and statistical analyses.

## Results

We identified 26,236 patients who met the initial inclusion criteria of a recent dental visit with antibiotic prescribed by a VA dentist within 7 days and had a prior, nonanaphylactic, penicillin allergy history. Of those, 25,661 (98%) received a noncephalosporin antibiotic. After stratified random sampling by geographic region, the cohort included 100 patients with a penicillin allergy who did not receive a cephalosporin (cohort 1) and 200 patients with a penicillin allergy who did receive a cephalosporin (cohort 2).

Penicillin allergic reactions for cohort 1 were noted to span from intolerances to high-risk categories upon chart review. Several patients had multiple reactions: 6 reactions were categorized as intolerances, 12 were considered low risk, 92 were moderate to high risk, and 1 was considered high risk (Table 1). When applying penicillin allergy algorithms based on patient allergy history, 53% of patients were eligible for penicillin skin testing, 27% of patients were eligible for either oral penicillin challenge or skin testing, and 1% was contraindicated from algorithm-based penicillin allergy testing because fever was identified on chart review and a high-risk reaction contraindicates any allergy testing. Overall, 19% of patients did not have a documented type of allergic reaction to penicillin, 10% had a pseudo penicillin allergy, and 71% had a true penicillin allergy. We detected significant associations between the penicillin allergy categories (true vs pseudo vs missing) and unknown, remote allergy history and rash.

Facility and patient characteristics for cohort 1 are presented in Table 2. Almost 20% of facilities were rural, and they had a wide range of complexity and US Census regions. Patients were predominately male (92%), white (70%), and aged >64 years (59%). Common medical comorbidities included prosthetic orthopedic implant (31%), cardiac condition (25%), chronic pulmonary disease (11%), and diabetes (11%). Most patients were from high-complexity facilities in urban areas (80%).

Dental visit data for cohort 1 is summarized in Table 3. Most visits did not involve an extraction (78%). Also, 52% of dental visits had an invasive procedure that involved manipulation of the gingiva. The most common procedure types were diagnostic (68%), followed by oral and maxillofacial surgery (24%). Most visits included a general dentist (79%) and most visits had a clindamycin prescription associated with that visit (83%), followed by amoxicillin (9%).

**Table 3.** Dental Visit and Procedure Characteristics for Penicillin Allergic Patients Who Did Not Receive a Cephalosporin

Variable	Overall (N = 100)	Reaction Not Specified In Chart/Missing (N = 19)	Pseudo Penicillin Allergy (N = 10)	True Penicillin Allergy (N = 71)	*P* Value
**Extraction type**					.5535^a^
No extraction	78 (78.0)	14 (73.7)	8 (80.0)	56 (78.9)	
Simple extraction	11 (11.0)	2 (10.5)	0 (0)	9 (12.7)	
Surgical extraction	11 (11.0)	3 (15.8)	2 (20.0)	6 (8.5)	
**Invasiveness**					.2491^a^
Routine	48 (48.0)	9 (47.4)	3 (30.0)	36 (50.7)	
Mildly invasive	14 (14.0)	2 (10.5)	4 (40.0)	8 (11.3)	
Invasive	38 (38.0)	8 (42.1)	3 (30.0)	27 (38.0)	
**Procedure type**					
Adjunct	9 (9.0)	1 (5.3)	2 (20.0)	6 (8.5)	.3733^a^
Diagnostic	68 (68.0)	13 (68.4)	5 (50.0)	50 (70.4)	.4495
Endodontics	5 (5.0)	2 (10.5)	0 (0)	3 (4.2)	.4376^a^
Implant services	6 (6.0)	0 (0)	0 (0)	6 (8.5)	.4982^a^
Maxillofacial prosthetics	0 (0)	0 (0)	0 (0)	0 (0)	…
Oral & maxillofacial surgery	24 (24.0)	6 (31.6)	2 (20.0)	16 (22.5)	.7477^a^
Orthodontics	0 (0)	0 (0)	0 (0)	0 (0)	…
Periodontics	11 (11.0)	1 (5.3)	1 (10.0)	9 (12.7)	.8715^a^
Preventive	4 (4.0)	1 (5.3)	0 (0)	3 (4.2)	1.0000^a^
Prosthodontics	3 (3.0)	1 (5.3)	0 (0)	2 (2.8)	.6465^a^
Prosthodontics, fixed	1 (1.0)	0 (0)	0 (0)	1 (1.4)	1.0000^a^
Restorative	15 (15.0)	4 (21.0)	5 (50.0)	6 (8.5)	.0036^a^
**Dentist subtype**					
General dentist	79 (79.0)	17 (89.5)	7 (70.0)	55 (77.5)	.4094^a^
Endodontist	2 (2.0)	0 (0)	0 (0)	2 (2.8)	1.0000^a^
Oral & maxillofacial	17 (17.0)	2 (10.5)	1 (10.0)	14 (19.7)	.6224^a^
Periodontist	4 (4.0)	0 (0)	2 (20.0)	2 (2.8)	.0698^a^
Prosthodontist	0 (0)	0 (0)	0 (0)	0 (0)	…
Other	7 (7.0)	0 (0)	1 (10.0)	6 (8.5)	.4326^a^
**Antibiotic associated with visit**					
Amoxicillin	9 (9.0)	5 (26.3)	1 (10.0)	3 (4.2)	.0148^a^
Clindamycin	83 (83.0)	12 (63.2)	8 (80.0)	63 (88.7)	.0284^a^
Cephalexin	0 (0)	0 (0)	0 (0)	0 (0)	…
Azithromycin	4 (4.0)	0 (0)	0 (0)	4 (5.6)	.7231^a^
Penicillin	3 (3.0)	1 (5.3)	1 (10.0)	1 (1.4)	.2009^a^
Doxycycline	2 (2.0)	1 (5.3)	0 (0)	1 (1.4)	.4980^a^
Metronidazole	0 (0)	0 (0)	0 (0)	0 (0)	…
Fluoroquinolone	0 (0)	0 (0)	0 (0)	0 (0)	…

Note. Statistical significance = *P* < .0007, Bonferroni corrected.

a Fisher exact test.

For cohort 2, factors associated with receipt of a cephalosporin versus other antibiotics are described in Table 4 and Table 5. The odds of receiving a cephalosporin were lower for male sex (vs female) (OR, 0.44; 95% CI, 0.20–0.99) and African American compared to White race (OR, 0.40; 95% CI, 0.21–0.76). Additionally, having an allergy to macrolides (OR, 3.12; 95% CI, 1.05–9.28; reference, no allergy to macrolides) or other antibiotics (non–β lactams, noncephalosporins, nonsulfas, nonmacrolides, nonquinolones; OR, 3.00; 95% CI, 1.45–6.21; reference, no allergy to other antibiotics) was associated with an increased odds of receiving a cephalosporin. A higher Elixhauser score was also associated with a decreased odds for receiving a cephalosporin (OR, 0.87; 95% CI, 0.76–0.98). Within 30 days of the antibiotic dispense date, 1 patient who received a cephalosporin had a nonanaphylactic allergic reaction. Even though patients were documented as penicillin allergic, 5% received a penicillin (defined as penicillin, ampicillin, amoxicillin, or amoxicillin/clavulanic acid).

**Table 4.** Patient Demographics and Facility Characteristics for Cohort 1 and Cohort 2

Variable	Overall (N = 300)	Received cephalosporin [Cohort 2] (N = 200)	Did not receive cephalosporin [Cohort 1] (N = 100)	*P* Value	Unadjusted OR (95% CI)
**Patient demographics and characteristics**					
**Sex**				.0433	
Female	41 (13.7)	33 (16.5)	8 (8.0)		Reference
Male	259 (86.3)	167 (83.5)	92 (92.0)		**0.44 (0.20–0.99)**
**Race**				.0267^a^	
White	231 (77.0)	161 (80.5)	70 (70.0)		Reference
African American	46 (15.3)	22 (11.0)	24 (24.0)		**0.40 (0.21–0.76)**
Other	7 (2.3)	6 (3.0)	1 (1.0)		2.61 (0.31–22.07)
Missing	16 (5.3)	11 (5.5)	5 (5.0)		0.96 (0.32–2.86)
**Age**				.5788	
18–44 y	27 (9.0)	17 (8.5)	10 (10.0)		Reference
45–64 y	105 (35.0)	74 (37.0)	31 (31.0)		1.40 (0.58–3.41)
>64 y	168 (56.0)	109 (54.5)	59 (59.0)		1.09 (0.47–2.53)
**Smoking**				.4108	
Current smoker	91 (30.3)	63 (31.5)	28 (28.0)		Reference
Never smoked	58 (19.3)	41 (20.5)	17 (17.0)		1.07 (0.52–2.20)
Past smoker	73 (24.3)	50 (25.0)	23 (23.0)		0.97 (0.50–1.88)
Missing	78 (26.0)	46 (23.0)	32 (32.0)		0.64 (0.34–1.20)
**Antibiotic allergies**					
β-lactams	4 (1.3)	3 (1.5)	1 (1.0)	1.00^a^	1.51 (0.16–14.68)
Cephalosporins	13 (4.3)	8 (4.0)	5 (5.0)	.7657^a^	0.79 (0.25–2.49)
Sulfas	48 (16.0)	34 (17.0)	14 (14.0)	.5040	1.26 (0.64–2.47)
Macrolides	27 (9.0)	23 (11.5)	4 (4.0)	.0324	**3.12 (1.05–9.28)**
Quinolones	33 (11.0)	24 (12.0)	9 (9.0)	.4337	1.38 (0.62–3.09)
Other	60 (20.0)	50 (25.0)	10 (10.0)	.0022	**3.00 (1.45–6.21)**
**Comorbidities**					
Cardiac condition	69 (23.0)	44 (22.0)	25 (25.0)	.5605	0.85 (0.48–1.49)
Prosthetic orthopedic implant	88 (29.3)	57 (28.5)	31 (31.0)	.6539	0.89 (0.53–1.50)
HIV-AIDS	0 (0)	0 (0)	0 (0)	…	…
Cancer	12 (4.0)	8 (4.0)	4 (4.0)	1.00^a^	1.00 (0.29–3.40)
Heart failure	14 (4.7)	7 (3.5)	7 (7.0)	.2438^a^	0.48 (0.16–1.41)
Chronic pulmonary disease	28 (9.3)	17 (8.5)	11 (11.0)	.4829	0.75 (0.34–1.67)
Cerebrovascular disease	6 (2.0)	4 (2.0)	2 (2.0)	1.00 ^a^	1.00 (0.18–5.56)
Dementia	6 (2.0)	2 (1.00)	4 (4.0)	.0979^a^	0.24 (0.05–1.35)
Diabetes	34 (11.3)	23 (11.5)	11 (11.0)	0.8975	1.05 (0.49–2.25)
Liver disease	8 (2.7)	4 (2.0)	4 (4.0)	.4477^a^	0.49 (0.12–2.00)
Metastatic tumor	1 (0.3)	1 (0.5)	0 (0)	1.00^a^	>999 (<0.001–>999)
Myocardial infarction	3 (1.0)	3 (1.5)	0 (0)	.5533^a^	>999 (<0.001–>999)
Paralysis	2 (0.7)	2 (1.0)	0 (0)	.5541^a^	>999 (<0.001–>999)
Peripheral vascular disease	6 (2.0)	4 (2.0)	2 (2.0)	1.00^a^	1.00 (0.18–5.56)
Renal disease	22 (7.3)	12 (6.0)	10 (10.0)	.2103	0.57 (0.24–1.38)
Rheumatic disease	3 (1.0)	2 (1.0)	1 (1.0)	1.00^a^	1.00 (0.09–11.16)
Peptic ulcer disease	1 (0.3)	0 (0)	1 (1.0)	.3333^a^	>999 (<0.001–>999)
Gagne score median, mean (SD)	1, 7 (12.5)	1, 6 (11.6)	3, 9 (14.0)	.1755	0.99 (0.97–1.01)
Charlson score median, mean (SD)	0, 1 (1.5)	0, 1 (1.4)	0, 1 (1.6)	.5297	0.95 (0.81–1.11)
Elixhauser median, mean (SD)	1, 2 (1.8)	1, 2 (1.7)	2, 2 (2.1)	.0378	**0.87 (0.76–0.98)**
**Facility characteristics**					
**Rural**				.3661^a^	
No	251 (83.7)	171 (85.5)	80 (80.0)		Reference
Yes	45 (15.0)	26 (13.0)	19 (19.0)		0.64 (0.34–1.22)
Missing	4 (1.3)	3 (1.5)	1 (1.0)		1.40 (0.14–13.70)
**Complexity**				.8377	
1a	114 (38.0)	76 (38.0)	38 (38.0)		Reference
1b	63 (21.0)	39 (19.5)	24 (24.0)		0.81 (0.43–1.54)
1c	48 (16.0)	32 (16.0)	16 (16.0)		1.00 (0.49–2.05)
2	24 (8.0)	16 (8.0)	8 (8.0)		1.00 (0.39–2.54)
3	51 (17.0)	37 (18.5)	14 (14.0)		1.32 (0.64–2.74)
**US Census region**				1.00	
Midwest	75 (25.0)	50 (25.0)	25 (25.0)		Reference
Northeast	75 (25.0)	50 (25.0)	25 (25.0)		1.00 (0.51–1.97)
South and other	75 (25.0)	50 (25.0)	25 (25.0)		1.00 (0.51–1.97)
West	75 (25.0)	50 (25.0)	25 (25.0)		1.00 (0.51–1.97)

Note. Significant results are shown in bold (*P* < .05).

a Fisher exact test.

**Table 5.** Dental Visit and Procedure Characteristics for Cohort 1 and Cohort 2

Variable	Overall (N = 300)	Received cephalosporin (N = 200)	Did not receive cephalosporin (N = 100)	*P* Value	Unadjusted OR (95% CI)
**Extraction type**				.9601	
No extraction	235 (78.3)	157 (78.5)	78 (78.0)		Reference
Simple extraction	34 (11.3)	23 (11.5)	11 (11.0)		1.04 (0.48–2.24)
Surgical extraction	31 (10.3)	20 (10.0)	11 (11.0)		0.90 (0.41–1.98)
**Invasiveness**				.6600	
Routine	139 (46.3)	91 (45.5)	48 (48.0)		Reference
Mildly invasive	37 (12.3)	23 (11.5)	14 (14.0)		0.87 (0.41–1.84)
Invasive	124 (41.3)	86 (43.0)	38 (38.0)		1.19 (0.71–2.00)
**Procedure type**					
Adjunct	32 (10.7)	23 (11.5)	9 (9.0)	.5084	1.31 (0.59–2.96)
Diagnostic	205 (68.3)	137 (68.5)	68 (68.0)	1.00	1.02 (0.61–1.71)
Endodontics	16 (5.3)	11 (5.5)	5 (5.0)	.8558	1.11 (0.37–3.27)
Implant services	26 (8.7)	20 (10.0)	6 (6.0)	.2457	1.74 (0.68–4.48)
Maxillofacial prosthetics	1 (0.3)	1 (0.5)	0 (0)	1.00 ^a^	>999 (<0.001–>999)
Oral & maxillofacial surgery	76 (25.3)	52 (26.0)	24 (24.0)	.7073	1.11 (0.64–1.94)
Orthodontics	0 (0)	0 (0)	0 (0)	…	…
Periodontics	24 (8.0)	13 (6.5)	11 (11.0)	.1756	0.56 (0.24–1.31)
Preventive	7 (2.3)	3 (1.5)	4 (4.0)	.2272^a^	0.37 (0.08–1.67)
Prosthodontics	13 (4.3)	10 (5.0)	3 (3.0)	.5548^a^	1.70 (0.46–6.32)
Prosthodontics fixed	4 (1.3)	3 (1.5)	2 (2.0)	1.00^a^	1.51 (0.16–14.68)
Restorative	35 (11.7)	20 (1.0)	15 (15.0)	.2035	0.63 (0.31–1.29)
**Dentist subtype**					
General dentist	244 (81.3)	165 (82.5)	79 (79.0)	.4633	1.25 (0.69–2.29)
Endodontist	6 (2.0)	4 (2.0)	2 (2.0)	1.00^a^	1.00 (0.18–5.56)
Oral & Maxillofacial	41 (13.7)	24 (12.0)	17 (17.0)	.2346	0.67 (0.34–1.31)
Periodontist	15 (5.0)	11 (5.5)	4 (4.0)	.5741	1.40 (0.43–4.50)
Prosthodontist	5 (1.7)	5 (2.5)	0 (0)	.1734^a^	>999 (<0.001–>999)
Other	23 (7.7)	16 (8.0)	7 (7.0)	.7589	1.16 (0.46–2.91)
**Antibiotic associated with visit**					
Amoxicillin	12 (4.0)	3 (1.5)	9 (9.0)	.0032^a^	**0.15 (0.04–0.58)**
Clindamycin	101 (33.7)	18 (9.0)	83 (83.0)	<.0001	**0.02 (0.01–0.04)**
Cephalexin	200 (66.7)	200 (100.0)	0 (0)	<.0001	>999 (<0.001–>999)
Azithromycin	7 (2.3)	3 (1.5)	4 (4.0)	.2272^a^	0.37 (0.08–1.67)
Penicillin	3 (1.0)	0 (0)	3 (3.0)	.0363^a^	<0.001 (<0.001–>999)
Doxycycline	2 (0.7)	0 (0)	2 (2.0)	.1104^a^	<0.001 (<0.001–>999)
Metronidazole	4 (1.3)	4 (2.0)	0 (0)	.3052^a^	>999 (<0.001–>999)
Fluoroquinolone	1 (0.3)	1 (0.5)	0 (0)	1.00^a^	>999 (<0.001–>999)
**Reaction after antibiotic prescription date**					
Antibiotic hypersensitivity within 14 d	0 (0)	0 (0)	0 (0)	…	…
Antibiotic hypersensitivity within 30 d	1 (0.3)	1 (0.5)	0 (0)	1.00 ^a^	>999 (<0.001–>999)
Anaphylaxis within 14 d	0 (0)	0 (0)	0 (0)	…	…
Anaphylaxis within 30 d	0 (0)	0 (0)	0 (0)	…	…

Note. Significant results are shown in bold (*P* < .05).

a Fisher exact test.

## Discussion

Current evidence supports penicillin allergy evaluations by emergency clinicians, internists, intensivists, pharmacists, and infectious diseases specialists, as well as part of antibiotic stewardship programs.^10–17^ With the high number of patients with a documented penicillin allergy, it is reasonable for dentists to conduct penicillin allergy evaluations.

In this study evaluating a sample of VA dental patients, our findings suggest that dental offices represent an opportunity for penicillin allergy evaluation because 10% of patients had a pseudo allergy. This rate is much higher than the 0.5%–2.0% of hypersensitivity reactions or nonallergic reported in the literature, but this lower rate may be a result of the VA population being older and having allergies that wane over time.^4,5,10^ Most nonanaphylactic penicillin allergies were rashes, all of which were eligible for skin testing or an oral test dose challenge based on evidence-based algorithms. However, 19% of patients did not have an allergic reaction documented. These results are consistent with the literature, which shows that rashes were a commonly documented reaction and that ∼20% of penicillin allergic patients do not have reaction documented.^9^

Interestingly, findings suggest that some dentists are already informally evaluating penicillin allergy status and making nuanced antibiotic prescribing decisions. This study identified potentially unsafe practices, such as dentists prescribing penicillin even in the setting of a true penicillin allergy. Thus, additional studies are needed to determine the true extent of such practices. Additionally, these practices further support usage of simple algorithms that could assist dentists with avoiding prescribing a penicillin-based antibiotic to a truly allergic patient and encouraging penicillin allergy assessment in others.

The exact role for dentists in a penicillin allergy program remains unclear. Dentists with a busy practice may not be interested in taking on additional clinical responsibilities, or they may have limited experience or training in conducting allergy evaluation. Furthermore, patient perception of penicillin allergy skin testing or an amoxicillin oral challenge at a dental office is unknown. Dentists may also be risk averse to performing an allergy assessment in a dental office, including fear of litigation. An alternate solution may be patient completion of a penicillin-allergy questionnaire in a dental office waiting room, online prior to appointment, or as a routine question on their written or online health history. The dentist could review the results and discuss next steps with the patient, including following up with a primary care provider or an allergist for further evaluation.

The involvement of dentists in identifying opportunities for assessment and the evaluation of penicillin allergies is an important step with the final goal of delabeling the inappropriate penicillin allergy. Penicillin allergy delabeling is an important part of antimicrobial stewardship because it would reduce the usage of second line (ie, clindamycin) or broad-spectrum antibiotics, both of which are associated with adverse outcomes such as MRSA, *C. difficile* infection, and surgical site infections.^8,9^

This study had several limitations. VA data generally include patients with lower socioeconomic status and older patients, and they may not be fully representative of penicillin allergic patients in non-VA settings. However, these results still provide a broad view of national opportunities. Analyses were based on electronic health record review; therefore, it is possible that some verbal information between patients and dentists may have occurred and were not documented in the chart. This may be particularly true in cases in which penicillin allergy information was missing. In this setting, it is possible that dentists had more information about the penicillin allergy than was available through chart review and CDW. Additionally, the proposed penicillin-allergy assessment algorithms used were designed primarily for medical settings.^10^ However, such an approach could be adapted for use in dental settings.

In conclusion, dental offices represent a unique opportunity for penicillin allergy assessment. This analysis reveals that most penicillin-allergic patients seen at VA dental clinics would be eligible for penicillin skin testing or an oral penicillin challenge and that opportunities exist for penicillin allergy delabeling. Further research is needed to understand the role of dentists and dental clinics in assessing penicillin allergies.

## Supplementary material

For supplementary material accompanying this paper visit https://doi.org/10.1017/ash.2022.18. click here to view supplementary material
