# Supplementary material for: Opportunities for penicillin allergy evaluation in dental clinics
Source: Antimicrob Steward Healthc Epidemiol. 2022 Apr 11;2(1):e58. doi: 10.1017/ash.2022.18 (PMC9726497; doi:10.1017/ash.2022.18)
Supplement: Supplementary file 1 [file S2732494X22000183sup001.docx]

Supplemental Table 1. Definition of penicillin allergy using ICD9/10-CM and E codes

| Allergic reaction | ICD-9 / ICD-10-CM | E Codes |
| --- | --- | --- |
| Dermatitis due to drug | 693.0 / L270 or L271 | E930 – Antibiotics causing adverse effects in therapeutic use  E931 – Other anti-infectives causing adverse effects in therapeutic use |
| Allergic urticaria | 708 / L50 |  |
| Angioneurotic edema | 995.1 / T78.3XXA |  |
| Anaphylaxis | 995.0 / T78.2XXA |  |

| Supplemental Table 2. Definitions for penicillin allergy histories: indicators for true allergy, and eligibility for oral challenge or skin testing | | | | |
| --- | --- | --- | --- | --- |
|  | Shenoy True Allergy | Penicillin testing contraindicated | Eligible for direct oral challenge for low risk patients | Eligible for penicillin skin test for low risk patients |
| *Penicillin Intolerance histories* |  |  |  |  |
| Isolated GI upset (diarrhea, nausea, vomiting, abdominal pain) | No | No | Yes | Yes |
| Chills (rigors) | No | No | Yes | Yes |
| Headache | No | No | Yes | Yes |
| Fatigue | No | No | Yes | Yes |
| Other | No | No | Yes | Yes |
| *Low risk allergy histories* |  |  |  |  |
| Family history | No | No | Yes | Yes |
| Itching (pruritus) | No | No | Yes | Yes |
| Unknown, remote (<10 year ago) | No | No | Yes | Yes |
| Patient denies allergy but is on record | No | No | Yes | Yes |
| *Moderate-high risk allergy histories* |  |  |  |  |
| Anaphylaxis | Yes | No | No | Yes |
| Cough | Yes | No | No | Yes |
| Throat tightness | Yes | No | No | Yes |
| Shortness of breath | Yes | No | No | Yes |
| Wheezing | Yes | No | No | Yes |
| Angioedema/swelling | Yes | No | No | Yes |
| Nasal symptoms | Yes | No | No | Yes |
| Hypotension | Yes | No | No | Yes |
| Rash | Yes | No | No | Yes |
| Dizzy/lightheadedness | Yes | No | No | Yes |
| Bronchospasm | Yes | No | No | Yes |
| Arrythmia | Yes | No | No | Yes |
| Flushing/redness | Yes | No | No | Yes |
| Syncope/pass out | Yes | No | No | Yes |
| *High risk histories* |  |  |  |  |
| Stevens-Johnson | Yes | Yes | No | No |
| Fever | Yes | Yes | No | No |
| Dystonia | Yes | Yes | No | No |
| Drug reaction eosinophilia | Yes | Yes | No | No |
| Serum sickness | Yes | Yes | No | No |
| Organ injury (liver/kidney) | Yes | Yes | No | No |
| Anemia | Yes | Yes | No | No |
| Thrombocytopenia | Yes | Yes | No | No |
| Erythema multiforme | Yes | Yes | No | No |
| Acute generalized exantematous | Yes | Yes | No | No |

This table was adapted from Shenoy et al. appendix toolkit.^10^ Pseudo allergy equates to intolerance and low risk allergy histories by Shenoy et al. Patients with missing allergy information could not be classified

Supplemental Figure 1.

Prior anaphylaxis to an antibiotic

Yes [n=946]

(excluded)

No [n=208,576]

No [n=182,340]

Yes [n=26,236]

Received cephalosporin

Yes [n=575]

No [n=25,661]

Chart reviewed:

100 patients

Chart reviewed:

200 patients

Antibiotic cohort (1/1/2015-12/31/2018): patients who were seen in OP dental clinics who also received an antibiotic prescription written by a dentist (doesn’t need to be the same provider) within 7 days of the visit (-7D to +7D). Those with oral infection in the previous 7 days were removed. [n=209,522]

Prior penicillin allergy
